# A global overview of genetically interpretable multimorbidities among common diseases in the UK Biobank

**DOI:** 10.1186/s13073-021-00927-6

**Published:** 2021-07-05

**Authors:** Guiying Dong, Jianfeng Feng, Fengzhu Sun, Jingqi Chen, Xing-Ming Zhao

**Affiliations:** 1 Institute of Science and Technology for Brain-inspired Intelligence, Fudan University, Shanghai, 200433 China; 2 MOE Key Laboratory of Computational Neuroscience and Brain-Inspired Intelligence, and MOE Frontiers Center for Brain Science, Fudan University, Shanghai, 200433 China; 3 Zhangjiang Fudan International Innovation Center, Shanghai, 200433 China; 4 Molecular and Computational Biology Program, University of Southern California, Los Angeles, CA 90089 USA

**Keywords:** Multimorbidity, Genetic factors, Converged biological function, Hub diseases, Multimorbidity module

## Abstract

**Background:**

Multimorbidities greatly increase the global health burdens, but the landscapes of their genetic risks have not been systematically investigated.

**Methods:**

We used the hospital inpatient data of 385,335 patients in the UK Biobank to investigate the multimorbid relations among 439 common diseases. Post-GWAS analyses were performed to identify multimorbidity shared genetic risks at the genomic loci, network, as well as overall genetic architecture levels. We conducted network decomposition for the networks of genetically interpretable multimorbidities to detect the hub diseases and the involved molecules and functions in each module.

**Results:**

In total, 11,285 multimorbidities among 439 common diseases were identified, and 46% of them were genetically interpretable at the loci, network, or overall genetic architecture levels. Multimorbidities affecting the same and different physiological systems displayed different patterns of the shared genetic components, with the former more likely to share loci-level genetic components while the latter more likely to share network-level genetic components. Moreover, both the loci- and network-level genetic components shared by multimorbidities converged on cell immunity, protein metabolism, and gene silencing. Furthermore, we found that the genetically interpretable multimorbidities tend to form network modules, mediated by hub diseases and featuring physiological categories. Finally, we showcased how hub diseases mediating the multimorbidity modules could help provide useful insights for the genetic contributors of multimorbidities.

**Conclusions:**

Our results provide a systematic resource for understanding the genetic predispositions of multimorbidities and indicate that hub diseases and converged molecules and functions may be the key for treating multimorbidities. We have created an online database that facilitates researchers and physicians to browse, search, or download these multimorbidities (https://multimorbidity.comp-sysbio.org).

**Supplementary Information:**

The online version contains supplementary material available at 10.1186/s13073-021-00927-6.

## Background

Multimorbidity, the coexistence of more than one disease in a patient not by chance, presents great challenges for disease diagnosis and treatment [1, 2]. Compared with single disease, multimorbidities are usually associated with more adverse health outcomes, such as lower life quality and higher mortality rate, and with higher economic burden [3–5]. Understanding the mechanisms of multimorbidities may be helpful for their early diagnosis, treatment, and management, thereby helping reduce the global disease burdens associated with multimorbidities.

During the last decade, large-scale genome-wide association studies (GWASs) have found overlapped genetic risks for a few frequently multimorbid diseases at the genomic loci level, i.e., single-nucleotide polymorphisms (SNPs) or genes, suggesting that there might be a molecular basis of multimorbidity. For example, GWASs have uncovered 38 SNPs associated with both asthma and allergic diseases [6], and 187 genome loci associated with at least two of ankylosing spondylitis, Crohn’s disease, psoriasis, primary sclerosing cholangitis, and ulcerative colitis [7]. Additionally, Sánchez-Valle et al. found that disease interactions inferred from similarities between patients’ gene expression profiles have significant overlaps with epidemiologically documented multimorbid relations [8], further supporting the genetic basis of multimorbidity. Moreover, several studies pointed out that diseases with higher probability of concurrency tend to share more associated genes [9, 10]. These findings have accumulated useful information to inform the biological etiology of multimorbidity [10, 11].

The malfunctions caused by disease risk loci can spread via cellular networks owing to molecular interactions among genes. To this end, some studies captured the genetic overlaps between multimorbidities by network-level evidence, such as protein-protein interactions (PPIs) and molecular pathways [9, 12, 13]. For example, Park et al. found a significantly positive correlation between the number of shared PPIs and the extent of disease concurrency by integrating information of cellular interactions, disease–gene associations, and Medicare data [9]. Moreover, a significantly increased number of shared pathways between cancers and multimorbid Mendelian diseases have also been observed [10]. These results indicate that dysfunctional entanglement in molecular networks might contribute to the existence of multimorbid diseases in patients. Additionally, some multimorbidities have also been reported to be similar in their overall genetic architectures measured by genetic correlations, such as the widespread genetic correlations among multimorbid psychiatric disorders [14–17].

Due to the limited access to the matched epidemiological and genomic data of the same population group, existing studies either used matched data for a limited number of diseases [6, 7, 11, 14–17], or collected large-scale genomic data and epidemiology data from different sources [9, 10]. However, the separation of genetic and epidemiological data makes it tricky to decide whether the shared genetic risks identified from one group can actually explain multimorbidity identified in another group. In the past few years, the UK Biobank (UKB) has collected hospital inpatient data and genetic data for about four hundred thousand individuals, providing a unique opportunity for investigating the genetics underlying multimorbid relationships among hundreds of common diseases [18].

In this study, we take advantage of the large-scale, matched epidemiological and genetic data hosted in the UKB to systematically investigate the multimorbid relationships among 439 common diseases as well as their shared genetic factors. We have identified the multimorbidity shared genetic components at the loci and network levels, and performed functional analyses on them to uncover the converged biological functions. Furthermore, the shared genetic patterns of the multimorbidities affecting the same and different physiological systems have been explored. Finally, we have constructed and decomposed two multimorbidity networks to find the hub diseases that mediate multimorbid relationships in multimorbidity modules and to highlight the corresponding molecular mechanisms. Our results provide a systematic resource of multimorbidities among common diseases, as well as their shared genetic risks (online database at https://multimorbidity.comp-sysbio.org). The converged biological molecules and functions identified in this study are responsible for many multimorbidities, which may serve as the key factors for the management and treatment of multimorbidities.

## Methods

### Population data

Population data used in this study is collected from the UKB [18]. More than 500,000 individuals aged 40–69 living in the UK were recruited to the assessment centers and signed an electronic consent to allow a broad range of access to their anonymized data for health-related research.

### Disease selection and classification

In the UKB, field-41270 is the summary diagnoses of 410,293 patients across all their hospital inpatient records, which are coded according to the International Classification of Disease version 10 (ICD10). For a detailed list of the ICD10 codes, see https://www.icd10data.com/. A total number of 11,727 ICD10 codes are recorded with affected patients. We define common diseases as the level 2 ICD10 codes (the three-character ICD10 codes used by the UKB) with prevalence > 0.1%, since most of the publicly available GWAS summary statistics of the UKB diagnoses are based on the level 2 codes [19, 20]. Patients diagnosed with codes at or under level 2 are considered as suffering from the corresponding level 2 diseases. Only the ICD10 codes within the range of chapter I ~ XIV are considered, which can be found on the website of the UKB (https://biobank.ndph.ox.ac.uk/showcase/field.cgi?id=41270). Due to the too-detailed phenotype descriptions of the ICD10 codes, such as F20 (Schizophrenia) and F25 (Schizoaffective disorders), we further aggregate the highly similar ICD10 codes into one disease according to the phecodes [21]. Phecode is a collection of manually curated phenotypes by experts with the advantage of better aligning with diseases mentioned in clinical and genomic research [21]. Finally, the diseases are manually classified, mostly according to their affected physiological systems while also considering their origins [22].

### Identification of multimorbidities

In this study, we define multimorbidities as disease pairs coexisting in one person not by chance [1]. For each disease pair (disease *i* and *j*), we quantify the relative risk (RR) between them by

1 $$ RR=\frac{N\times {C}_{ij}}{I_i\times {I}_j}, $$

where *N* denotes the total number of patients (i.e., 410,293) recorded in field-41270 (summary diagnoses); *I*_*i*_(*I*_*j*_) denotes the number of patients who suffered from disease *i*(*j*); *C*_*ij*_ denotes the number of patients who were diagnosed with both disease *i* and *j* [9]. As *RR* is a monotonically increasing function of *C*_*ij*_, the one-sided *P* value is equal to the sum of the probabilities when *C*_*ij*_ is greater than or equal to the actual value. Therefore, we calculate the *P* value by approximating the binomial distribution as a Poisson distribution

2 $$ P=\overset{N}{\underset{k={C}_{ij}}{\varSigma\ }}\frac{\mathit{\exp}\left(-{C}_{ij}^{\ast}\right)\times {\left({C}_{ij}^{\ast}\right)}^k}{k!}, $$

where $$ {C}_{ij}^{\ast }={I}_i{I}_j/N $$.

Field-41280 in the UKB records the diagnosis date corresponding to each diagnosis in field-41270. We denote *C*_*simul*(*ij*)_ as the number of patients who were diagnosed with both disease *i* and *j* at the same date. The diagnosis summary data in the UKB only includes 410,309 participants, which is relatively small when compared with other electronic health record datasets (usually more than tens of millions) [9, 23, 24]. Besides, we argue that the more often two diseases are diagnosed within 1 day, the more likely they will be multimorbid (Additional file 1: Supplementary Methods and Results). Therefore, to get a more credible collection of multimorbidities, only disease pairs with a high proportion of patients diagnosed within 1 day are considered, namely, disease pairs with *C*_*simul*(*ij*)_/*I*_*i*_ > 1% or *C*_*simul*(*ij*)_/*I*_*j*_ > 1%. Then, disease pairs with *RR* > 1 and *P* value <0.05/ the total number of disease pairs with RR > 1 are selected as multimorbidities (Bonferroni correction).

### Validation of the multimorbidities in the UKB

We compare the multimorbidities identified by us to that identified by Jensen et al. [25], Blair et al. [24], and Hidalgo et al. [23]. Jensen et al. studied the disease trajectories and found 4014 significantly directional multimorbidities among 681 level 2 ICD10 diseases [25]. We extract the diseases used by both us and Jensen et al. [25]. We take all disease pairs among the commonly used diseases as background, and use Fisher exact test to examine the significance of overlaps of multimorbidities in the UKB and Jensen et al [25]. Blair et al. reported 2909 multimorbid relationships among 95 Mendelian diseases and 65 complex diseases in over 110 million patients [24]. One Mendelian or complex diseases correspond to one or more ICD10 codes. We treat the ICD10 codes below level 2 as their corresponding level 2 ICD10 diseases. We extract the diseases that are used by both us and Blair et al. [24]. Fisher exact test is used to examine the significance of multimorbidity overlaps between us and Blair et al. [24], with all disease pairs between the commonly used Mendelian and complex ICD10 diseases as background. Hidalgo et al. constructed a Phenotypic Disease Network based on 995 three-digit ICD9 codes [23]. The ICD9 codes can be found in https://www.icd9data.com. For comparison, the three-digit ICD9 codes are mapped to the level 2 ICD10 codes by Unified Medical Language System (UMLS) [26]. We download the MetamorphoSys software from UMLS and install the UMLS Knowledge Sources of ICD9CM and ICD10 metathesaurus [26]. Then, we construct the mappings between the ICD9 and ICD10 codes through their common UMLS identifiers. We consider four types of mapping relationships: (1) the three-digit ICD9 codes can be directly mapped to the level 2 ICD10 codes; (2) the three-digit ICD9 codes can be mapped to the child nodes of the level 2 ICD10 codes; (3) the child nodes of the three-digit ICD9 codes can be mapped to the level 2 ICD10 codes; (4) the child nodes of the three-digit ICD9 codes can be mapped to the child nodes of the level 2 ICD10 codes. For the last three types, the mapping relationships of the child nodes are considered as the mappings of their parent three-digit ICD9 codes and level 2 ICD10 codes. The resulted mappings are many-to-many. In total, 786 three-digit ICD9 codes from Hidalgo et al. [23] can be mapped to 1230 level 2 ICD10 codes. Since one ICD9 code may map to multiple ICD10 codes, the two ICD9 codes of one multimorbidity from Hidalgo et al. [23] may map to two sets of level 2 ICD10 codes. We treat any combinations of diseases between the resulted two ICD10 sets as multimorbidities of Hidalgo et al. We extract the commonly used diseases by us and Hidalgo et al. [23]. Fisher exact test is used to examine the significance of multimorbidity overlaps between us and Hidalgo et al. [23] with all disease pairs between the commonly used diseases as background. Since not all disease pairs provided by Hidalgo et al. [23] are multimorbidities, we compare our multimorbidities with two sets of disease connections from Hidalgo et al.: one set including diseases-pairs with RR > 1 and primary *P* values < 0.05, the other set including diseases-pairs with RR > 1 and FDR-corrected *P* values < 0.05.

### Multimorbidity tendency of intra- and inter-categories

To investigate whether there are pairs of categories (including the same category combination) wherein the diseases in one category tend to be multimorbid with diseases in the other category, we firstly construct a multimorbidity network where the nodes represent diseases and edges represent the multimorbid relationships between disease pairs. We then randomly shuffle the node labels in the network while keeping the structure of the network unchanged, and repeat the randomization process 10^6^ times. For each randomized multimorbidity network, we count the number of multimorbid relationships (*L*_*mn*_) between category *m* and *n*, and calculate an empirical *P* value between each pair of categories as follows:

3 $$ P=\frac{\sum \limits_{i=1}^{10^6}\ {Ind}_i}{10^6}, $$

where *Ind*_*i*_ is an indicator function, with value 1 when ${L}_{mn}\ge {L}_{mn}^{\ast }$ and value 0 when ${L}_{mn}<{L}_{mn}^{\ast }$. ${L}_{mn}^{\ast }$ is the actual number of multimorbidities between category *m* and *n* in the original multimorbidity network.

### GeneAtlas of the UKB

Canela-Xandri et al. published a batch of GWAS summary statistics including > 30 million genetic variants associated with 778 traits of 452,264 UKB participants (http://geneatlas.roslin.ed.ac.uk) [19]. Out of the 778 traits, 657 were binary phenotypes generated from multiple fields in the UKB including ICD10 codes from hospital diagnoses (field-41202 and field-41204, which are the primary and secondary diagnoses recorded in field-41207), ICD10 codes from cancer register (field-40006), self-reported diseases (field-20002), and 3 other traits. We choose the GWAS summary statistics of the level 2 ICD10 codes to explore the genetic basis of multimorbidities. The detailed quality controls of the GWAS are described in the original publication. We further remove variants with minor allele frequency (MAF) < 0.01, HEW < 1e−50, and INFO score < 0.9. The remained 7,423,311 variants are used in our analysis.

### Shared genetic components of multimorbidities

We dissect the shared genetic components of multimorbidities from five aspects, i.e., SNP, gene, PPI, pathway, and the overall genetic architecture.

#### SNP

For each disease, we firstly select the SNPs with *P* values < 0.05 / (variant counts × disease counts) from the GWAS summary statistics as significant, and then expand these significant SNPs to their linkage disequilibrium (LD) blocks with LD score *r*^2^ > 0.8 and LD window 1 MB. The reference LD scores between SNPs are calculated by plink (--ld-window 100 --ld-window-kb 1000) [27] based on individuals of European ancestry from the 1000 Genomes Project (GRCh37) [28]. We take the LD expanded SNPs as disease-associated SNPs and calculate the multimorbidity shared SNPs as those associated with both diseases of the multimorbidity. Fisher exact test is used to test the significance of SNP overlaps between multimorbidities with the 7,423,311 variants used in this study as background.

#### Gene

We identify disease-associated genes from the GWAS summary statistics by three methods—direct mapping, eQTL, and MAGMA [29]. (1) Direct mapping: the disease-associated SNPs are mapped to genes based on genomic coordinates of genes annotated in GRCh37.p13 (within 2 kb upstream and 500 bp downstream of gene body). The gene location file is obtained from NCBI website (https://www.ncbi.nlm.nih.gov, GRCh37.p13). (2) eQTL: the identified loci of GWAS may exert their effects on phenotypes via regulating gene expressions, so we leverage the eQTL data from GTEx (https://gtexportal.org/home/datasets, v7) to find genes whose expressions are related to the disease-associated SNPs (adjusted *P* value<0.05, FDR corrected) [30]. (3) MAGMA: MAGMA is a tool for aggregating multiple genetic markers in gene based or gene-set based ways to estimate their joint effects on phenotypes, based on GWAS summary statistics or individual genotype data [29]. We identify genes by aggregating the effects of SNPs by MAGMA based on GWAS summary statistics. The SNPs are mapped to genes according the gene location file (build 37) provided by the MAGMA website (https://ctg.cncr.nl/software/magma), and the window size is set to 2 kb upstream and 0.5 kb downstream. The European panel of the 1000 Genomes is used as reference data to estimate LD between SNPs [28]. For the SNPs of synonym, only the first one is reserved according to the synonym file in the MAGMA website. We define the multimorbidity shared genes as those associated with both diseases of the multimorbidity. Fisher exact test is used to test the significance of gene overlaps between multimorbidities with all human genes as background.

#### PPI

We download PPIs from BioGRID [31] and select 41,980 human physical PPIs for analysis. For each PPI (consisting gene1 and gene2) and multimorbidity combination, if gene1 is associated with one disease of the multimorbidity and gene2 is associated with the other disease of the multimorbidity, we consider this PPI as the multimorbidity shared PPI. For a PPI, if one of its genes is related to both diseases of the multimorbidity, we do not consider it as the multimorbidity shared PPI. To test the significance of PPI overlaps of multimorbidities, we randomly select the disease genes from all human genes while keeping the number of disease genes unchanged, and then identify the multimorbidity shared PPIs with the same method. We repeat this process 10^4^ times and calculate the *P* value as the frequency of the random number equal to or greater than the real number of shared PPIs.

#### Pathway

MSigDB database compiles 1329 canonical pathways, where several pathways are extremely large (Additional file 2: Fig. S1) [32]. We remove 29 KEGG disease pathways and 43 large pathways (6 KEGG, 5 NABA, and 32 REACTOME pathways) with more than 200 genes. The remained 1257 pathways are used for enrichment analysis of disease-associated genes. Fisher exact test is used to determine the enrichment *P* values. Pathways with adjusted *P* values < 0.05 (FDR corrected) are considered as associated with disease. We define the multimorbidity shared pathways as those enriched by the gene set of one disease and containing at least one gene of the other disease. To test the significance of pathway overlaps of multimorbidities, we randomly select the disease genes from all human genes while keeping the number of disease genes unchanged, and then identify the multimorbidity shared pathways with the same method. We repeat this process 10^4^ times and calculate the *P* value as the frequency of the random number equal to or greater than the real number of shared pathways.

#### Overall genetic architecture

The genetic architecture similarity between disease pair is measured by the genetic correlation. We estimate the genetic correlation of liability scale from GWAS summary statistics by LD score regression (LDSC) [33]. LDSC evaluates the genetic correlation between two diseases from GWAS summary statistics based on the fact that the effect size of a given SNP incorporates the effects of all SNPs in its LD region [34]. SNPs with high LD will have higher *χ*^2^ statistics on average than those with low LD. A similar relationship remains if the *χ*^2^ statistics for a single study are replaced by the product of the *Z* scores from two studies of traits with nonzero genetic correlation [33, 34]. LDSC is not biased by sample overlap and is fast to compute. The LD score computed from 1000 Genomes European data is used as reference [28], which is suitable for the GWAS summary data of the UKB population.

### Genetic association pattern of multimorbidities

To explore the genetic association pattern of multimorbidities at specific levels of genetic components, i.e., SNP, gene, PPI, pathway, or genetic correlation, we focus on the multimorbidities with genetic information available. For each type of genetic components, we permutate its interpretable multimorbidities and non-interpretable multimorbidities, and then compare the number of the interpretable multimorbidities after permutation (*N*_*perm*_) to the actual number (*N*_*real*_) between any pair of categories (including between the same categories). We repeat this permutation 10^5^ times and calculate *P* value as the frequency of *N*_*perm*_ ≥ *N*_*real*_. *P* values are adjusted by FDR method for multiple hypothesis testing. Through this process, we can find category pairs where the multimorbidities are significantly interpreted by SNP, gene, PPI, pathway, and genetic correlation, respectively.

### Comparison with Park et al

Park et al. have reported 2239 disease pairs with shared genes, PPIs, or co-expressed genes [9]. We use Fisher exact test to examine whether these 2239 disease pairs are significantly overlapped with the genetically interpretable multimorbidities identified in our analysis. To perform Fisher exact test, we use the 8212 UKB multimorbidities with available genetic information as background. As the diseases used by Park et al. is coded by ICD9CM, we mapped ICD9 codes to ICD10 codes according to UMLS [26].

### Functional analysis of SNPs

We examine the function properties of SNPs from three aspects—genome-wide distribution, deleteriousness, and impacts on splicing. (1) We annotate the SNPs to genomic categories by ANNOVAR [35]. The categories are further merged into three groups—genic (including exonic, intronic, and splicing), noncoding RNA, and intergenic. (2) The deleteriousness of SNPs is quantified by CADD scores [36]. (3) The impacts of SNPs on splicing are quantified by dbscSNC scores [37]. For each genomic category, we perform Fisher exact test to examine whether the multimorbidity SNPs (SNPs shared by multimorbidities) are enriched in this category by taking the disease SNPs (SNPs associated with at least one of the 439 diseases) and SNPs used in this study (7,423,311) as background, respectively. *t* test is used to evaluate whether the CADD and dbscSNC scores of multimorbidity SNPs are different from those of other disease SNPs (SNPs associated with diseases but not associated with multimorbidities), and non-disease SNPs (all SNPs used in this study that are not associated with diseases).

### Functional analysis of genes

We explore the function properties of genes from three aspects—essentiality, pLI, and active tissue. (1) To predict the essentiality of human genes, we use the genotype-phenotype information in the Mouse Genome Informatics (http://www.informatics.jax.org). One human gene is considered essential if the knockout of its ortholog gene of mouse confers risks of lethality. We firstly extract the mouse phenotypes related to embryonic, prenatal, and postnatal lethality and identify the mouse gene list associated with these phenotypes. Then, the genes without knockout experiment supporting for lethality are dropped from this gene list. Finally, we take the human orthologous genes corresponding to these mouse genes as the human essential genes. Fisher exact test is used to evaluate whether multimorbidity genes (gene associated with multimorbidities) or disease genes (genes associated with at least one of the 439 diseases) are enriched in human essential genes by taking all human genes as background. (2) The pLIs of human genes are obtained from the Exome Aggregation Consortium (ExAC) [38]. We use *t* test to compare the pLIs of multimorbidity genes with that of other disease genes (genes associated with diseases but not associated with multimorbidities), and non-disease genes (all human genes that are not associated with diseases), respectively. (3) To count the number of active tissues per gene, we obtain the gene expression profiles for 53 tissues from GTEx v.7 [30]. For one gene, we define it as active in a given tissue if the average transcripts per kilobase million is > 1. We then group genes into 4 categories, that is, genes expressed in single tissue, 2–5 tissues, 6–42 tissues, 43–53 tissues. Mann–Whitney U test (two-sided) is used to evaluate whether the numbers of active tissues of multimorbidity genes are different from that of other disease genes and non-disease genes.

### GO enrichment analysis

We obtain 7530 gene sets related to GO biological processes from MSigDB (https://www.gsea-msigdb.org/gsea/msigdb/index.jsp) [32]. GO terms with more than 200 genes are removed, and the remained 6631 GO terms are used for the following enrichment analysis. Fisher exact test is used to identify enriched GO terms. FDR is used for multiple testing correction, and GO terms with adjusted *P* values < 0.05 are considered as significant.

### Small worldness of multimorbidity network

To evaluate the small worldness of the multimorbidity network, we firstly investigate whether the distribution of the node degrees in the network obeys the power law distribution. We fit the node degrees to power law distribution and obtain the parameters *α* and *X*_min_. If 2 < *α* < 3, we select the nodes with degrees > *X*_min_ to fit the log relationship between the degree and the number of nodes by Ordinary Least Square (OLS). If the fitting goodness (*Adj. R*^*2*^) of OLS > 0.1 and *P* value < 0.05, we consider the degree distribution of the network obeys the power law distribution. Secondly, we calculate the small-world coefficient (*sigma*) by comparing the average clustering coefficient and shortest path length of the network against the same quantities of its equivalent random networks. If the network is with *sigma* > 1 and degrees following power law distribution, we consider it has the property of small worldness.

### Identification of multimorbidity modules

We use the Louvain algorithm to detect the modules in the multimorbidity network [39]. Louvain algorithm is a heuristic method based on modularity optimization. It has good quality in community detection and works in a computationally fast way. When detect modules, we set the parameter resolution to 1, as this parameter produces the largest modularity (Q).

## Results

### Multimorbidities among common diseases in the UKB

In total, 439 common diseases (prevalence > 0.1%) are selected for the multimorbidity analysis from the UKB hospital inpatient data (see “Methods;” Additional file 3: Table S1), covering 385,335 patients. The average age of the patients at their first hospital diagnosis is 54, and there are more female patients than male patients (55% VS. 45%).

**Fig. 1:**
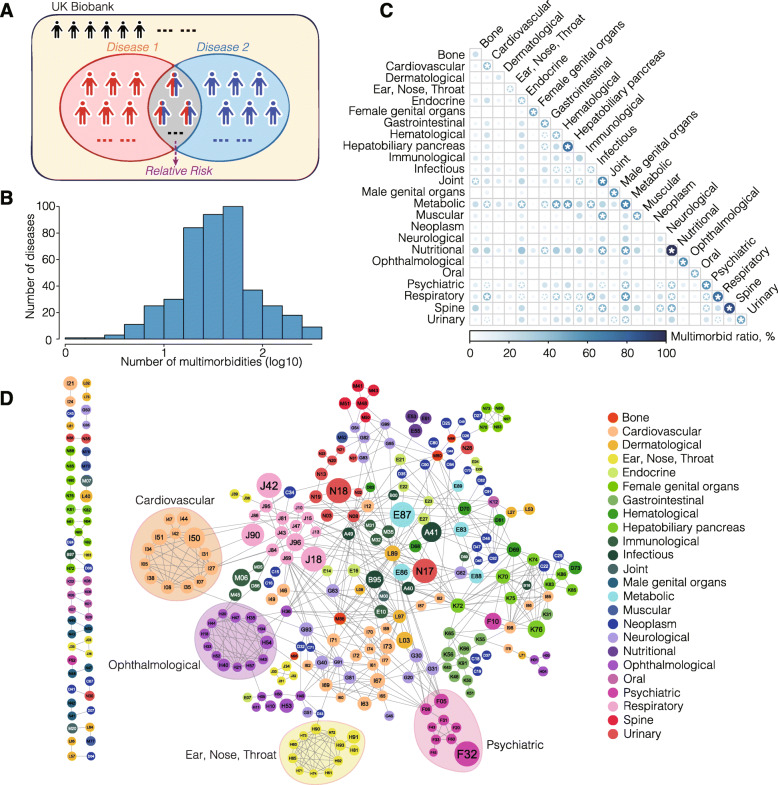
Multimorbidities identified in the UKB. **a** Schematic illustration of how RR is calculated for each pair of diseases. **b** The distribution of the number of multimorbidities for the UKB diseases. **c** Multimorbidity tendency of intra- and inter-physiological categories. Color and size of the circles represent the proportions of multimorbidities in all disease pairs within a category or between two categories. The deeper the color and the larger the size of a circle, the higher the proportion is. Star represents adjusted *P* value < 0.05 (FDR corrected). **d** The high-confidence multimorbidity network constructed by only including multimorbidities with RR > 15. Each node represents a disease, and each edge represents a multimorbid relationship between two diseases. The color code of a node represents the category of the disease. The size of each node is proportional to the number of its multimorbidities (not restricted to RR > 15)

In all, 11,285 multimorbidities are identified, involving 438 out of the 439 diseases (RR > 1, *P* value < 4.1e−6 with Bonferroni correction for all disease pairs with RR > 1, Fig. 1a, Additional file 3: Table S2; see “Methods”), with D21 (other benign neoplasms of connective and other soft tissue) being the only exception. Most diseases have fewer than 100 multimorbid partners (average of 51, Fig. 1b). We observe that diseases with high prevalence tend to have more multimorbid partners (Pearson’s correlation, *r* = 0.69, *P* value = 3.3e−64, Additional file 2: Fig. S2). For example, the top three diseases with the most multimorbid partners—hypertension (I10), hyperlipidemia (E78), and type 2 diabetes (E11), have prevalence of 27.5%, 13.1%, and 7.1% in the UKB, respectively.

To validate the credibility of the multimorbid relationships found through our analysis, we compare them to the multimorbid relationships identified by Jensen et al. [25], Blair et al. [24], and Hidalgo et al. [23]. Jensen et al. reported 4014 significantly directional multimorbidities between 681 diseases [25]. In total, 384 diseases are commonly used by us and Jensen et al. (Table 1) [25]*.* The comparison results show that the multimorbidities identified by us and Jensen et al. [25] have a significant overlap (*OR* = 6.8, *P* value = 0, Fisher exact test). We confirm a relatively high proportion (49%) of Jensen’s multimorbidities, compared to 13% of our results confirmed by Jensen et al. [25]. This might be because Jensen et al. [25] only provided the directional multimorbidities and ignored the multimorbidities without directions. When we only compare the directional multimorbidities in the UKB to Jensen et al. [25], the overlap is still significant (OR = 14.6, *P* value = 4.0e−212), and the proportion of the UKB multimorbidities confirmed by Jensen et al. increases to 32%. Studying the directionality of multimorbidities requires a very large sample size and a long follow-up time. The diagnosis data in the UKB has a long follow-up time (25 years, compared to 15 years by Jensen et al. [25]), but a relatively small sample size (about 0.4 million, compared to 6.2 million by Jensen et al. [25]). Despite this, the comparison with Jensen et al. [25] confirms the reliability of our multimorbidities. Blair et al. reported 2909 significant multimorbidities between 95 Mendelian diseases and 65 complex diseases [24]. Among these diseases, 14 Mendelian diseases and 62 complex diseases are commonly used by us and Blair et al*.* (Table 1) [24]. The overlap of multimorbidities in the UKB and Blair et al. [24] is also highly significant (OR = 3.1, *P* value = 1.8e−7). Hidalgo et al. constructed a Phenotypic Disease Network using the medical claims of more than 30 million patients [23]. This network contains the RRs among 995 three-digit ICD9 diseases as well as the corresponding *P* values. The ICD9 codes are mapped to ICD10 codes by the UMLS (see “Methods”) [26]. A total of 397 ICD10 diseases are commonly used by us and Hidalgo et al. (Table 1) [23]. The comparison shows that our multimorbidities have significant overlaps with the highly possible multimorbidities of Hidalgo et al. (*P* values = 2.3e−189 and 4.6e−187, based on original and FDR corrected *P* values, respectively) [23]. Taken together, we consider that our results can be confirmed by previous results and are of high reliability. Importantly, benefiting from the long follow-up time (25 years) and the extensive coverage of diseases provided by the UKB hospital inpatient data, the collection of multimorbidities reported here is the largest one for common disease multimorbidities by far, and thus provides an atlas of multimorbidities valuable for further analysis.

**Table 1:** Multimorbidity comparison with Jensen et al., Blair et al., and Hidalgo et al.

	Shared diseases	Multimorbidities in the UKB^a^	Multimorbidities in the reference^a^	Overlapping multimorbidities	Odds ratio	*P* values
Jensen et al. [25]	384	10,144	2595	1278	6.8	0
Jensen et al. [25]	384	989 (directional)	2595	318	14.6	4.0e−212
Blair et al. [24]	62 complex diseases, 14 Mendelian diseases	175	622	152	3.1	1.8e−7
Hidalgo et al. (RR > 1 and original P values < 0.05) [23]	397	8791	54,542	7242	2.2	2.3e−189
Hidalgo et al. (RR > 1 and FDR corrected P values < 0.05) [23]	397	8791	54,528	7234	2.2	4.6e−187

^a^The “Multimorbidities in the UKB” and “Multimorbidities in the reference” denote the multimorbidities formed by the shared diseases in the results of the UKB and reference, respectively

### Prevalent multimorbidities of intra- and inter-physiological systems

In the classic Human Disease Network (HDN) by Goh et al., diseases are connected if they share at least one disease-associated gene [22]. We have observed several clusters in HDN formed by diseases affecting the same physiological systems, such as cardiovascular diseases and nutritional diseases. This inspires us to explore whether diseases affecting the same physiological system tend to be multimorbidities. We divide the 439 diseases into 24 categories, mostly according to their affected physiological systems while also considering their origins (e.g., “Neoplasm”) (Additional file 3: Table S1), and calculate the disease multimorbidity tendency among the 24 categories (see “Methods”). As expected, we find significantly prevalent multimorbidities within 19 out of 24 categories (Fig. 1c). For example, 89% (32/36) of all disease pairs within the “Spine” category are multimorbidities (adjusted *P* value = 5e−5), and all the 4 nutritional diseases are multimorbid with one another (adjusted *P* value = 8.9e−3). To get a visual and more confident observation, we have constructed a high-confidence multimorbidity network comprised of multimorbidities with RR > 15 (Fig. 1d) and observed small and large clusters of multimorbidities affecting the same physiological systems, many of which are supported by previous studies—for examples, multimorbidity clusters affecting the “Cardiovascular” [40], the “Ophthalmological” [41], the “Ear, Nose, Throat” [42], and the “Psychiatric” [43] categories. These findings suggest the existence of shared mechanisms for diseases affecting the same physiological systems.

Interesting, apart from the intra-category multimorbidities, we also find significantly more multimorbid relationships between 41 pairs of different categories (Fig. 1c). Diseases from the “Respiratory” category are significantly more likely to be multimorbid with diseases from 11 other categories, followed by diseases from the “Metabolic” category which tend to coexist with diseases from 10 other categories, both suggesting the existence of shared etiologies beyond the boundaries of physiological systems. Moreover, metabolic diseases have the overall highest rate of inter-category multimobidities, which is consistent with many reports on the involvement of altered metabolisms in a wide range of diseases [44, 45]. It is noteworthy that sometimes the significant inter-category multimorbidity patterns are mediated by a small number of diseases that have a large number of multimorbid partners. For example, in the high-confidence multimorbidity network (Fig. 1d), psychiatric disorders are multimorbid with neurological disorders predominantly through F05 (delirium, not induced by alcohol and other psychoactive substances) and F06 (other mental disorders due to brain damage and dysfunction and to physical disease). In fact, for 33 out of the 41 significant category pairs, more than 50% of the inter-category multimorbid relationships are mediated through no more than three “hub” diseases. Take one of the most centered hubs as an example, E66-Obesity mediates more than half of the multimorbid relationships between the “Nutritional” category and three other categories—“psychiatric,” “spine,” and “joint.” This is consistent with previous findings that obesity is usually associated with mental, joint, and spinal diseases, such as depressive disorders, anxiety disorders, gout, and spondylosis [46–48]. As a result, understanding the mechanisms underlying multimorbidities, especially those mediated by the “hub” diseases, may provide a way forward to understand how they happen, and to seek to manage or treat them simultaneously.

### 46% of the multimorbidities are genetically interpretable

Previous studies have shown that disease pairs sharing more genes or PPIs are more likely to be multimorbidities [9, 10]. However, it still remains unclear that how many multimorbidities share genetic components (deemed as genetically interpretable), and whether there are any specific patterns in the shared genetic components for different types of multimorbidities. To explore these two questions, we capture the genetic associations of multimorbidities at 3 levels, i.e., the loci level (SNP and gene), the network level (PPI and pathway), and the overall genetic architecture level (genetic correlation) (see “Methods”).

**Fig. 2:**
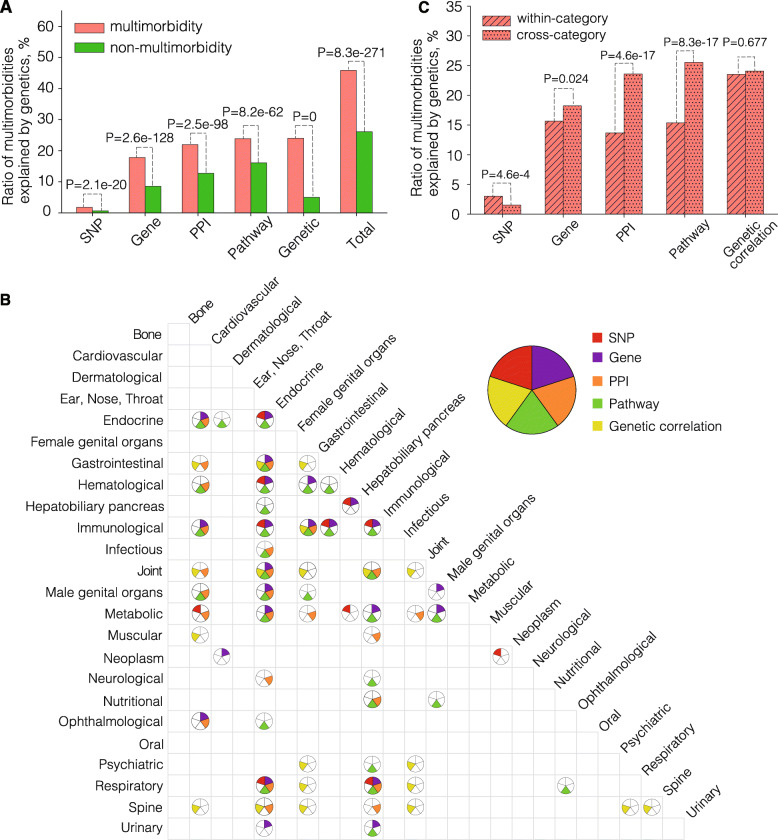
Multimorbidities interpreted by genetic components. **a** Ratios of multimorbidities and non-multimorbidities interpretable through SNP, gene, PPI, pathway, and genetic correlation. **b** Intra- and inter-category multimorbidities that can be significantly interpreted through the five types of genetic components. Each circle is divided into five parts, representing the five types of genetic components. Color-filled parts of each circle represent the types of genetic components that can significantly interpret the corresponding intra- or inter-category multimorbidities. No circle is drawn where none of the five types of genetic components is significant. **c** Ratios of multimorbidities of intra- and inter-categories interpretable through SNP, gene, PPI, pathway, and genetic correlation

All available GWAS summary statistics based on the UKB subjects are collected from geneAtlas [19], covering 332 out of the 439 diseases used in this study and comprising 8212 multimorbidities. We find 46% (3766) of these multimorbidities have shared genetic components: 147, 1463, 1803, and 1959 multimorbidities share SNPs, genes, PPIs, and pathways, respectively; and 1970 multimorbidities have significant genetic correlations (Fig. 2a, Additional file 3: Tables S3, S4, S5, S6, S7, see “Methods”). Multimorbidities are significantly more likely to share genetic components, compared with non-multimorbidities, across all genetic levels as well as their aggregation (Fig. 2a, see “Methods”). Additionally, we also find that 98%, 70%, and 100% of the genetically interpretable multimorbidities share significantly more SNPs, genes, and pathways than expected, respectively (see “Methods”). Only 5% of multimorbidities share significantly more PPIs than expected, possibly due to the incompleteness of PPI collections. Moreover, the genetically interpretable multimorbidities have a significant overlap with disease pairs reported by Park et al., which have shared genes, PPIs, or co-expressed genes (*P* value = 2.4e−6, Fisher exact test), further confirming our findings [9]. As the genetic information and the epidemiological information come from the same subjects, we consider our results relatively robust against the usual confounding factors for the genetic analysis of multimorbidities, such as differences in genetic background. Thus, our results strongly support the existence of genetic predispositions for almost half of the multimorbid relationships. We have created an online database to facilitate researchers and physicians to browse, search or download the multimorbidities (https://multimorbidity.comp-sysbio.org).

We then explore whether there are any specific patterns in shared genetic components for different types of multimorbidities, i.e., exploring the differences in the levels of genetic components mediating intra- and inter-category multimorbidities. Overall, we find that multimorbidities of 9 out of 24 (37.5%) intra-categories and 52 out of 276 (18.8%) inter-categories significantly share genetic components, suggesting a relatively high probability of genetic involvement for multimorbidities affecting the same physiological systems (Fig. 2b). Interestingly, as in Fig. 2b and c, intra-category multimorbidities are slightly more likely to share loci-level genetic components (*P* value = 4.6e−4 for SNPs; difference not significant for genes after Bonferroni correction; Fisher exact test) compared to inter-category multimorbidities, while the latter are more likely to share network-level genetic components (*P* values = 4.6e−17 and 8.3e−17 for PPIs and pathways, respectively). There is no significant difference in how likely multimorbidities of intra- and inter-categories have genetic correlations. These results suggest that multimorbidities affecting the same and different physiological systems may have different biological origins—the former tends to directly originate from pleiotropic loci and the latter tends to indirectly originate from converged biological functions.

The above statistical observation is best illustrated by the diseases from “Male genital organs” category (Fig. 2b). The multimorbidities within the “Male genital organs” category tend to share genes (adjusted *P* value = 4.1e−2, FDR corrected). In fact, 50% (8/16) of the multimorbidities within this category share disease-associated genes. A total of 20 genes are involved in these intra-category multimorbid relationships and are mainly related to the human leukocyte antigen (HLA) complex (such as *HLA-DQA1* and *HLA-DRB1*), histone clusters (such as *HIST1H1B* and *HIST1H2AJ*), and tumors (such as *TERT* and *NOTCH4* for prostate cancer) [49, 50]. In contrast, multimorbid relationships of inter-categories involving the “Male genital organs” category tend to share pathways (72/142, 51%). The KEGG pathway “cell adhesion molecules cams” is shared by half (36/72) of these inter-category multimorbid relationships, followed by “antigen processing and presentation,” which is shared by 32 of these multimorbidities.

To summarize, based on matched genetic and epidemiological data, we find that almost half (46%) of the multimorbidities identified in this study are genetically interpretable, indicating a strong genetic role in the origin of multimorbidities. Among these genetically interpretable multimorbidities, the intra-category and inter-category ones tend to share genetic components at different levels (loci VS. network), suggesting their different biology origins.

### Genetically interpretable multimorbidities converge on cell immunity, protein metabolism, and gene silencing

To enhance the understanding on the biological mechanisms of multimorbidities, we conduct functional analyses of the loci and network-level genetic components. The overall genetic architecture is not analyzed here, as it reflects statistical correlations but not detailed functions (see “Discussion”).

**Fig. 3:**
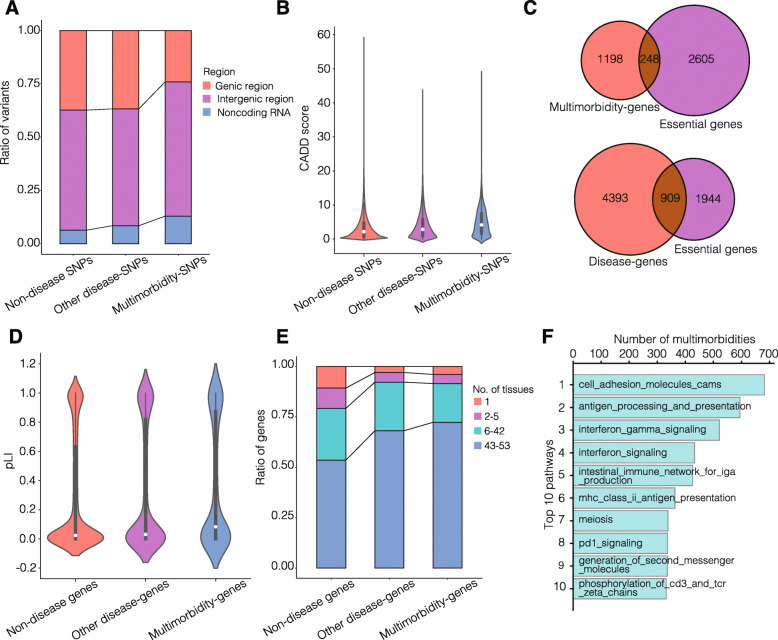
Characteristics of the genetic components shared by multimorbidities. **a** The ratios of SNPs located in genic region, intergenic region, and noncoding RNA region for multimorbidity SNPs, other disease SNPs, and non-disease SNPs. **b** CADD score distributions for multimorbidity SNPs, other disease SNPs, and non-disease SNPs. **c** Overlaps between multimorbidity genes and essential genes, and between disease genes and essential genes. **d** The pLI distributions of multimorbidity genes, other disease genes, and non-disease genes. **e** The ratios of genes expressed in certain numbers of tissues, for multimorbidity genes, other disease genes, and non-disease genes. **f** Top ten pathways that are shared by the largest numbers of multimorbidities

We firstly examine the genome-wide distribution and the deleteriousness of the multimorbidity SNPs (see “Methods”). We find that multimorbidity SNPs tend to be located in noncoding RNA (*P* values = 1.2e−44 and 4.1e−147) and intergenic regions (*P* values = 1.7e−54 and 2.9e−48) (Fig. 3a), but with slightly higher CADD scores (*P* values = 9.8e−129 and 0) than other disease SNPs and non-disease SNPs (see “Methods”; Fig. 3b). These results suggest that multimorbidity SNPs are slightly more deleterious, possibly through playing important roles in gene transcriptional regulations [51]. We have also examined the effects of multimorbidity SNPs on splicing by the dbscSNC splicing scores [37], but found no difference among the multimorbidity SNPs, other disease SNPs and non-disease SNPs (Additional file 2: Fig. S3). Additionally, we find that 73% of the multimorbidity SNPs locate in a small region of the genome—the HLA region (chr6:29,691,116–33,054,976), and 51% of the multimorbidities interpretable through SNPs share at least one HLA-region SNP. The HLA region is well known for its high degree, long-ranged LD blocks, which may help explain the pleiotropy of these SNPs in multimorbidities [52]. SNPs in this region have been previously predicted to be relevant for multiple autoimmune diseases through disrupting the regulation of immune-related genes [51]. Consistent with this, most of the top multimorbidities with the largest number of shared HLA-region SNPs involve autoimmune diseases or diseases with a significant autoimmune-related origin, such as E03 (Other hypothyroidism), J45 (Asthma), K90 (Intestinal malabsorption), E10 (Insulin-dependent diabetes mellitus), and G35 (Multiple sclerosis). Finally, we test whether the genomic location and the CADD score distributions of multimorbidity SNPs are mainly determined by the HLA-region variants. After removing the HLA-region SNPs, multimorbidity SNPs are still overrepresented in noncoding RNA regions (*P* values = 3.5e−47 and 1.2e−61) and still have significantly higher CADD scores (*P* values = 0.02 and 1.5e−15) than other disease SNPs and non-disease SNPs, but are no longer overrepresented in intergenic regions (Additional file 2: Figs. S4A, S4B). We conclude that multimorbidity SNPs, no matter whether in the HLA region or not, are slightly more deleterious and more likely to locate in noncoding RNA regions than other SNPs.

Goh et al. reported that most (78%) disease genes recorded in Online Mendelian Inheritance in Man are not essential genes critical for survival, and these disease genes are less likely to be housekeeping genes that express in all tissues [22]. We then test whether our multimorbidity genes behave similarly or differently. Here, we obtain 2852 essential genes, which are human orthologs of mouse genes whose disruptions are embryonically or postnatally lethal (see “Methods”). We find that only 17% of the disease genes and 17% of the multimorbidity genes are essential genes (Fig. 3c), although the disease genes and the multimorbidity genes are more enriched in essential genes (*P* values = 2.9e−38 and 8.8e−15, respectively). Essential genes are reported to have a tendency to encode hub proteins in the human interactome and play important roles in maintaining normal developmental and/or physiological functions [22]. These results indicate that most multimorbidity genes are functionally peripheral in the human interactome, and their mutations are compatible with survival into reproductive years so that these multimorbidity phenotypes are preserved in a population. Although most multimorbidity genes are not essential genes, we observe a higher probability of loss of function intolerances (pLIs) for multimorbidity genes, compared to other disease genes as well as non-disease genes (*P* values = 0.01 and 3.6e−11, respectively, *t* test; Fig. 3d). Removing the essential genes, this trend remains unchanged, suggesting that the higher pLIs distribution of multimorbidity genes is not just due to the essential genes (Additional file 2: Fig. S5). To examine whether multimorbidity genes tend to be housekeeping genes, we summarize the number of tissues each gene is expressed in based on the gene expression data of 53 tissues in GTEx [30]. We find that multimorbidity genes tend to be expressed in more tissues, compared to other disease genes and non-disease genes (*P* values = 4.7e−4 and 8.1e−44, respectively, two-sided Mann–Whitney U test; Fig. 3e). Considering the high pleiotropy of HLA regions, we recalculate the properties of the multimorbidity genes after removing the HLA variants. In this case, we find that multimorbidity genes are still mostly nonessential, and they still tend to have higher pLIs (*P* values = 0.04 and 2.9e−10) and express in more tissues (*P* values = 4.4e−3 and 1.2e−32) compared with other disease genes and non-disease genes (Additional file 2: Figs. S4C, S4D, S4E). As a result, we consider that multimorbidity genes are important for normal biological mechanisms, though most of them are not essential for survival, and disrupted multimorbidity genes may have clinical consequences affecting slightly more tissues than other genes.

For the network-level genetic components, the genes involved in the top 10 PPIs shared by the most numbers of multimorbidities are significantly enriched in GO terms related to biological processes of gene silencing and protein metabolism (localization, acetylation, ubiquitination, and catabolism) (see “Methods”). These 10 PPIs account for 18% of the multimorbidities interpretable through PPIs. Moreover, as shown in Fig. 3f, the top 10 pathways shared by the largest numbers of multimorbidities are predominantly immune-related processes and correspond to 56% of the multimorbidities interpretable through pathways. Most of the multimorbidities interpretable through these 10 top pathways are autoimmune or inflammatory diseases, such as J45 (Asthma), K20 (Oesophagitis), M06 (Other rheumatoid arthritis), L40 (Psoriasis), and E10 (Insulin-dependent diabetes mellitus). The findings based on the network-level genetic components suggest a phenomenon that a significant portion of genetically interpretable multimorbidities may converge on a handful of biological mechanisms, with the most common mechanisms related to cell immunity, gene silencing, and protein metabolism. This phenomenon is further supported by the loci-level genetic components: the top 10 genes can interpret as much as 41% of the gene interpretable multimorbidities and are enriched in immune-related GOs, such as “interferon gamma mediated signaling pathway,” “antigen processing and presentation of peptide antigen,” and “regulation of T cell mediated cytotoxicity.” Moreover, after removing HLA-region SNPs, we still observe that a few genetic components can interpret many multimorbidities (the top 10 SNPs, genes, PPIs, and pathways can interpret 23%, 30%, 15%, and 34% of multimorbidities interpretable through SNP, gene, PPI, and pathway, respectively). The top enriched GO terms by the genes and PPIs are “protein localization to chromosome telomeric region” and “beta-catenin-tcf complex assembly,” and the top enriched pathways are “rna pol i rna pol iii and mitochondrial transcription” and “meiosis” (Additional file 2: Fig. S4F).

### “Hub” disease-mediated genetically interpretable multimorbidity modules

The fact that a small number of genetic components can interpret a large portion of the genetically interpretable multimorbidities, inspires us to examine whether the “small world” property exists in these genetically interpretable multimorbidities. Therefore, we construct two multimorbidity networks by connecting multimorbid diseases that share the loci-level genetic components (denoted as the LG network) and the network-level genetic components (denoted as the NG network), respectively. As expected, for the LG and NG networks, their node degrees follow the power law distribution (Additional file 2: Fig. S6), and they have the attributes of small worldness based on the average clustering coefficients and average shortest path lengths (*sigma* = 1.15 and 1.03, respectively; see “Methods”). In previous sections, we have shown that many of the inter-category multimorbidities are mediated through hub diseases. Given the “small world” properties of the LG and NG networks, we hypothesize that there are multimorbidity modules in the two networks, possibly featured by hub diseases and specific genetic components. In order to test this hypothesis, we perform network decomposition to detect multimorbidity modules by the Louvain algorithm [39].

**Fig. 4:**
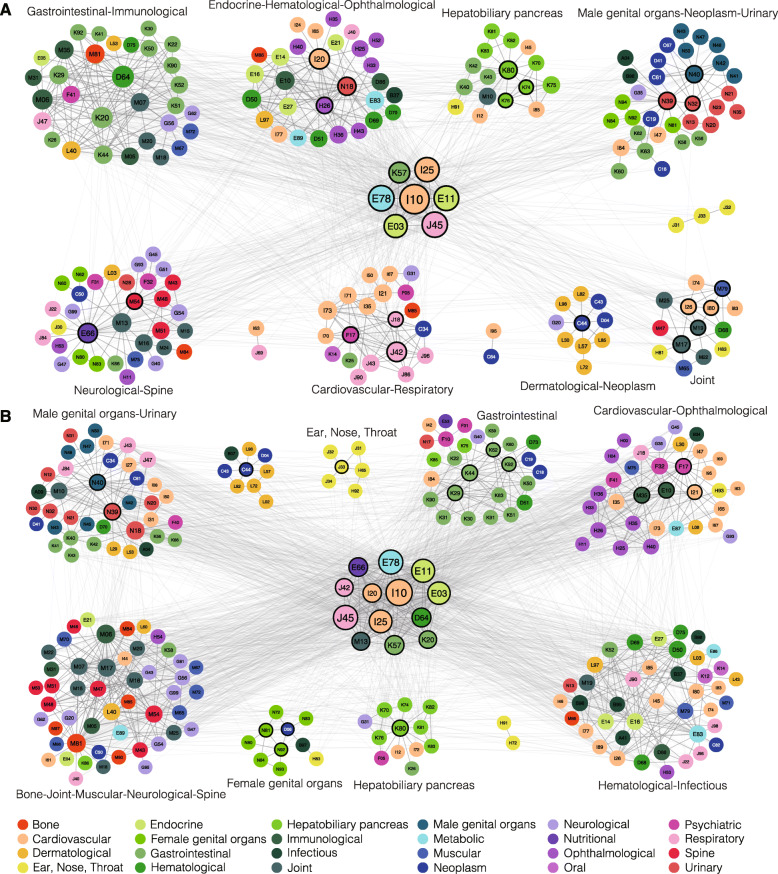
Loci-level (**a**) and network-level (**b**) genetically interpretable multimorbidity networks. **a, b** Each circle represents a disease. Colors of the circles (diseases) correspond to the categories of the diseases. “Universal hub diseases” are located at the center of each network, and diseases that belong to the same multimorbidity module are grouped close to each other. For each module, the featured categories are annotated. The black borders of the nodes indicate that they are the local hub diseases of the multimorbidity modules. Diseases that belong to neither universal hub diseases nor a multimorbidity module are not included in this figure

We first arbitrarily define nodes (diseases) connected with more than 25% of all nodes in each network as “universal hub diseases.” It is not appropriate to assign the “universal hub diseases” into any single multimorbidity module, as each module usually contains far fewer than 25% of all nodes. Seven and thirteen “universal hub diseases” are found for the LG network and the NG network, respectively (Fig. 4, Additional file 3: Table S8). These “universal hub diseases” are usually known to co-occur with many diseases. For example, I10 (Essential (primary) hypertension) connect to 235 (80%) diseases in the NG network and is well known for having heavy multimorbidity burdens [53].

Next, based on the remained nodes that are not “universal hub diseases,” we have detected 11 multimorbidity modules for the LG network and 10 multimorbidity modules for the NG network (Modularity *Q* = 0.40 and 0.32, respectively) (Fig. 4a, b, Additional file 3: Tables S9, S10). Overall, the module sizes (number of nodes in a module) range from 2 to 49, and the LG and NG networks have 8 and 9 multimorbidity modules whose sizes are larger than 5, respectively. Each multimorbidity module is assigned with “featured categories,” which are the categories that the diseases in this module significantly overrepresent (Fisher exact test, adjusted *P* values < 0.05, FDR corrected). Within each module (size > 5), if more than half of the within-module edges can be mediated by three or less top-degree diseases, we define them as “local hub diseases” of the module. As shown in Table 2, we have identified “featured categories” for all multimorbidity modules in the LG and NG networks, and “local hub diseases” for 7 and 7 multimorbidity modules in the LG and NG networks, respectively. We find that most local hub diseases belong to the featured categories of their modules, highlighting the prevalence of genetically interpretable multimorbidities within the same physiological system. Nonetheless, most modules have more than one featured category, showing that genetically interpretable multimorbidities are not limited by physiological boundaries.

**Table 2:** Genetically interpretable multimorbidity modules and their hub diseases

Multimorbidity module^a^	Module size	Featured categories	Hub diseases
LG-module1	33	Neurological-Spine	Obesity (E66) | Dorsalgia (M54)
LG-module2	33	Male genital organs-Neoplasm-Urinary	Hyperplasia of prostate (N40) | Other disorders of urinary system (N39) | Other disorders of bladder (N32)
LG-module3	31	Gastrointestinal-Immunological	/
LG-module4	29	Endocrine-Hematological-Ophthalmological	Angina pectoris (I20) | Chronic renal failure (N18) | Pulmonary embolism (H26)
LG-module5	20	Cardiovascular-Respiratory	Mental and behavioral disorders due to use of tobacco (F17) | Other chronic obstructive pulmonary disease (J42;J44) | Pneumonia, organism unspecified (J18)
LG-module6	16	Hepatobiliary pancreas	Cholelithiasis (K80) | Fibrosis and cirrhosis of liver (K74) | Other diseases of liver (K76)
LG-module7	14	Joint	Phlebitis and thrombophlebitis (I80) | Other arthrosis (M19) | Gonarthrosis [arthrosis of knee] (M17) | Other soft tissue disorders, not elsewhere classified (M79) | Pulmonary embolism (I26)
LG-module8	10	Dermatological-Neoplasm	Other malignant neoplasms of skin (C44)
NG-module1	49	Bone-Joint-Muscular-Neurological-Spine	/
NG-module2	41	Hematological-Infectious	/
NG-module3	39	Male genital organs-Urinary	Hyperplasia of prostate (N40) | Other disorders of urinary system (N39)
NG-module4	33	Cardiovascular-Ophthalmological	Mental and behavioral disorders due to use of tobacco (F17) | Insulin-dependent diabetes mellitus (E10) | Acute myocardial infarction (I21;I22) | Other systemic involvement of connective tissue (M35)
NG-module5	27	Gastrointestinal	Diaphragmatic hernia (K44) | Gastritis and duodenitis (K29) | Other diseases of digestive system (K92) | Other diseases of anus and rectum (K62)
NG-module6	12	Hepatobiliary pancreas	Cholelithiasis (K80)
NG-module7	10	Female genital organs	Carcinoma in situ of cervix uteri (D06;N87) | Excessive, frequent and irregular menstruation (N92) | Female genital prolapse (N81)
NG-module8	9	Dermatological-Neoplasm	Other malignant neoplasms of skin (C44)
NG-module9	6	Ear, Nose, Throat	Nasal polyp (J33)

^a^The “LG-module” and “NG-module” denote the multimorbidity modules identified by the Louvain algorithm in the LG-network and NG-network, respectively. The LG-network is constructed by connecting the multimorbid diseases that share the loci-level genetic components, and the NG-network is constructed by connecting the multimorbid diseases that share the network-level genetic components

**Fig. 5:**
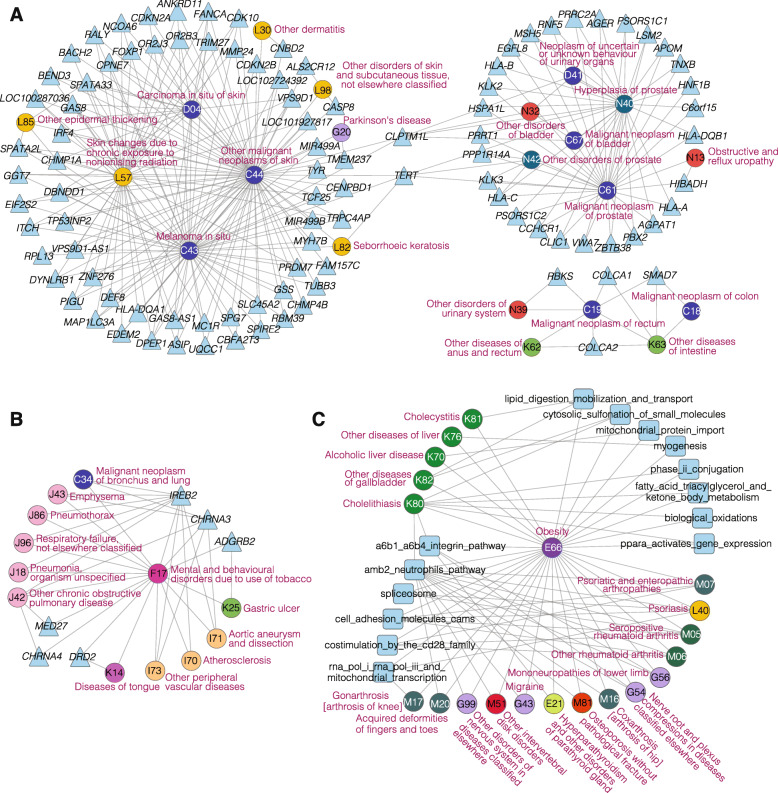
Case studies for genetically interpretable multimorbidity networks. **a–c** Circles, triangles, and squares represent diseases, genes, and pathways, respectively. Colors of the circles (diseases) correspond to the categories of the diseases, following the same color codes as in Fig. 1. **a** Multimorbidities of neoplasms and their shared genes in LG-module2 and LG-module8. **b** Multimorbidities of the hub disease F17 (Mental and behavioral disorders due to use of tobacco) in LG-module5, and their shared genes. **c** Pathways shared by the universal hub disease E66 (Obesity) and its multimorbidities in NG-module1 and NG-module6. Only the top 5 pathways shared by the largest numbers of multimorbidities are shown

We next describe several cases to illustrate how the network and module structures can help us understand the genetics underlying the large numbers of multimorbid relationships. First, some categories are consistently grouped together in modules. In both networks, we identify modules that feature the “Male genital organs-Urinary,” the “Dermatological-Neoplasm,” and the “Neurological-Spine” categories, confirming the genetic associations between the involved multimorbidities from multiple genetic levels (Table 2). Second, modules can help distinguish different multimorbidity tendencies and the corresponding genetic mechanisms among diseases of the same category. In the LG network, the “Neoplasm” category is in two modules—LG-module2 (“Male genital organs-Neoplasm-Urinary”) and LG-module8 (“Dermatological-Neoplasm”). Neoplasm diseases in LG-module2 are mainly prostate (C61), bladder (C67), urinary organs (D41), and intestinal (C18, C19;C20) cancers, while neoplasm diseases in LG-module8 are all skin cancers (C43, C44, D04) (Fig. 5a, Additional file 3: Table S9). Except for *TERT* and *CLPTM1L*, genes shared by neoplasm diseases and other diseases in the two modules are different, reflecting diverse mechanisms underlying neoplasms of different tissues and their multimorbidities. Third, hub diseases may provide a new perspective for understanding multimorbidities among diseases from different categories. For example, as shown in Fig. 5b, the “Psychiatric” disorder F17 (Mental and behavioral disorders due to use of tobacco) is a hub disease of LG-module5 (“Cardiovascular-Respiratory”) and is multimorbid with 5 respiratory diseases (emphysema (J43), pyothorax (J86;J93), respiratory failure (J96), chronic obstructive pulmonary disease (COPD, J42;J44), pneumonia (J18)), 3 cardiovascular diseases (atherosclerosis (I70), aortic aneurysm and dissection (I71), peripheral vascular diseases (I73)), and neoplasm disease of lung cancer (C34). The most common genes shared by these multimorbidities are *IREB2* and *CHRNA3*, located in 15q25, a well-known region for association with COPD, lung cancer, and smoking [54]. *IREB2* encodes an iron-responsive element-binding protein (IRP) that regulates the iron metabolism [54]. *CHRNA3* encodes the neuronal nicotinic acetylcholine receptor, and its mutation is associated with lung function and COPD severity in ever-smokers [55]*.* Though there are many other possible pathways individually associated with the above diseases, our analysis indicates that iron metabolism and the neuronal nicotinic acetylcholine receptor pathways may be the top candidates to examine when study the multimorbidities of these diseases with F17. Lastly, “universal hub diseases” connect to multiple modules, sometimes through different genetic components. In the NG network, the universal hub disease E66 (Obesity) have multiple connections to NG-module1 (“Bone-Joint-Muscular-Neurological-Spine”) and NG-module6 (“Hepatobiliary pancreas”) (Fig. 4b). Pathways shared by obesity and “Hepatobiliary pancreas” diseases are mostly related to the lipoprotein metabolism, such as “lipid digestion mobilization and transport,” “fatty acid triacylglycerol ketone body metabolism,” “cytosolic sulfonation of small molecules,” and “mitochondrial protein import,” while also related to biological oxidations, myogenesis etc. (Fig. 5c). In comparison, pathways shared by obesity and “Bone, Joint, Muscular, Neurological, Spine” diseases from NG-module1 are mostly related to immunity, cell adhesion, and transcription. In summary, the genetically interpretable networks and modules can provide insights through hub diseases for understanding the molecular mechanisms underlying multimorbidities and may help prioritize target genes and pathways for designing new treatment.

## Discussion

In this study, we have profiled the multimorbid relationships among the common diseases in the UKB and systematically investigated the genetic risks shared by multimorbidities. We report an atlas of 11,285 multimorbid disease pairs among 438 common diseases, which is by far the largest in scale. We find that 46% of the multimorbidities with available genetic information share genetic components in at least one of the three levels—loci, network, or overall genetic architecture, and show that multimorbidities affecting the same and different physiological systems tend to share different levels of genetic components. Functional analyses show that the loci-level genetic components shared by multimorbidities tend to be deleterious (for SNPs) and affect multiple tissues (for genes), and both loci- and network-level genetic components mainly converge on cell immunity, protein metabolism, and gene silencing related functions. We have also constructed two multimorbidity networks of genetically interpretable multimorbidities and show that hub diseases mediating the majority of within-module connections can provide useful insights into the genetic contributors for multimorbidities affecting different physiological systems. Therefore, our results provide a detailed multimorbid and genetic landscapes of common diseases, which may be valuable for guiding the early diagnosis, management, and treatment of multimorbidities.

Our results highlight shared genetic predispositions or mechanisms underlying multimorbidities, which may provide useful information for drug discovery. Theoretically, it is plausible to repurpose existing drugs that target the shared genetic components of a pair of multimorbid diseases, to treat the multimorbidity of the two diseases. In an exploratory test, we are able to identify 8458 drug-multimorbidity relationships where the drugs are known to target the multimorbidity genes (Additional file 3: Table S11). Interestingly, some of these drugs have been indeed used in the population with the corresponding multimorbidities. For example, the gene *EDNRA*, a known target of aspirin, is shared by the multimorbidity of I20 (Angina pectoris) and I25(Chronic ischaemic heart disease), and we find that 65% of the people suffering from both diseases report usage of aspirin in the UKB. Moreover, the indication of aspirin for I20 (Angina pectoris) and I25 (Chronic ischaemic heart disease) individually have been reported by the Comparative Toxicogenomics Database (CTD; http://ctdbase.org/; Additional file 3: Table S11) [56]. Besides this encouraging case, we also find a surprising case concerning Lansoprazole, a drug that targets *MAPT*, a gene shared by the multimorbidity of E66 (Obesity) and J84 (Other interstitial pulmonary diseases). 16.7% of the people suffering from both diseases used lansoprazole according to the UKB. We find the indications of lansoprazole only include E66 (Obesity) in CTD (Additional file 3: Table S11) [56], but lansoprazole was reported to be able to induce interstitial lung disease [57], suggesting that some patients with the multimorbidity of E66 (Obesity) and J84 (Other interstitial pulmonary diseases) could be due to the use of lansoprazole. Though very preliminary, these initial results shed light on the possibility that our resource of multimorbidities and their shared genetic components may help with drug discovery as well as avoid severe side-effects for treating multimorbidities in the future.

We have found a significant correlation between the number of genes associated with a disease and the number of its multimorbidities (*r* = 0.44, *P* value = 2.4e−17, Pearson correlation). Notably, as much as 1970 out of the 8218 (24%) multimorbidities have significant genetic correlations, supporting the polygenic architecture of complex diseases, while nearly half of them cannot be readily interpretable at either the loci or the network level (Additional file 2: Fig. S7). This indicates unidentified genetic information within the genetic architecture that may require further investigation, such as the copy number variants (CNVs). CNVs are the structural chromosomal variants greater than 1 kb in size, and usually have dosage effects on genes. Several common diseases have been reported to be associated with rare CNVs, such as autism and schizophrenia [58, 59]. In addition, we find that genetic correlation is positively and significantly correlated with RR (r = 0.39, *P* value = 1.9e−72, Additional file 2: Fig. S8A), and with phenotype similarity of multimorbid diseases (r = 0.22, *P* value = 0.03, Additional file 2: Fig. S8B, phenotype similarity pre-calculated by van Driel et al. [60]). Phenotype similarity and RR also have a positive and significant correlation (r = 0.27, *P* value = 1.5e-8, Additional file 2: Fig. S8C). These results suggest that genetic architecture might interpret multimorbidities by contributing to similarities in symptoms.

One possible limitation with our study is the sample size. Though the overall sample size is not small, there are not many cases for each disease or each multimorbid disease pair. As such, we may fail to identify some multimorbid disease pairs that are disproportionally represented in our dataset. Also, the GWAS analyses might miss variants with very small effects. Nevertheless, our study is the first and largest study that combines the epidemiological and genetic information of the same subjects to explore the genetic components underlying multimorbidities. The matched phenotype and genotype data makes our results less affected by population-related confounding factors. Based on our current findings, one interesting future direction is to integrate more samples from other studies and incorporate more types of data, such as the medical images and the quantitative traits, in order to analyze the endophenotypes that bridge disease pairs and deepen our understanding of the mechanisms of multimorbidities.

## Conclusions

In summary, we have performed, for the first time, a systematic analysis of multimorbid relations among common diseases as well as their shared genetic components based on the matched epidemiological and genetic data of the same subjects from the UKB. Our results illustrate the multimorbidity tendency and the genetic association patterns of multimorbidities of intra- and inter-physiological systems and indicate that the hub diseases and converged biological molecules and functions may be the key for managing multimorbidities. We have created an online database that facilitates researchers and physicians to browse, search, or download these multimorbidities (https://multimorbidity.comp-sysbio.org).

## Supplementary Information

**Additional file 1:.** Supplementary Methods and Results.

**Additional file 2: Supplementary Figures**. Size distributions of the Reactome pathways, KEGG pathways, BioCarta pathways and PID pathways (top). Size distributions of disease and multimorbidity pathways (reactome and non-reactome) when only using pathways with size <= 200 (bottom) (Fig. S1). Correlation between the prevalence of diseases and the number of multimorbidities (Fig. S2). Splicing score of multimorbidity SNPs, other disease SNPs and non-disease SNPs (Fig. S3). Characteristics of the genetic components shared by multimorbidities when removing HLA-region variants (Fig. S4). pLIs of multimorbidity genes, other disease genes and non-disease genes when removing essential genes (Fig. S5). Degree distributions of diseases in multimorbidity networks that share loci and network level genetic components (Fig. S6). Multimorbidity overlaps interpreted by SNPs, genes, PPIs, pathways and genetic correlations (Fig. S7). Correlations among genetic correlation (rg), relative risk (RR), and phenotype similarity (PheSim) of multimorbidities (Fig. S8). Number of multimorbidities with time windows of 1 day, 0.5 year, 1 year, 2 years, 3 years, 4 years, 5 years and with no time limit (Fig. S9). Multimorbidity tendency of intra- and inter-categories based on the new chapters (NC) of diseases (Fig. S10).

**Additional file 3: Supplementary Tables**. Common disease summary information (Table S1). Multimorbidities among common diseases in the UK Biobank (Table S2). Multimorbidities interpreted by SNPs (Table S3). Multimorbidities interpreted by genes (Table S4). Multimorbidities interpreted by PPIs (Table S5). Multimorbidities interpreted by pathways (Table S6). Multimorbidities interpreted by genetic correlations (Table S7). Summary of universal hub diseases in the LG- and NG-networks (Table S8). Multimorbidity modules in the LG-network (Table S9). Multimorbidity modules in the NG-network (Table S10). Multimorbidity-drug-prescription relations derived from multimorbidity genes, drug targets and the UK Biobank self-report medications (Table S11). Multimorbidities among all diseases (including both common and rare diseases) in the UK Biobank (Table S12). Multimorbidity comparison with Jensen *et al*. [25] and Blair *et al*. [24] at different time windows (Table S13).

## Data Availability

The epidemiological data used in the present study is available from the UK Biobank with restrictions applied. Data were used under license and thus are not publicly available. Access to the UK Biobank data can be requested through a standard protocol (https://www.ukbiobank.ac.uk/register-apply/). GWAS summary statistics used in this study can be accessed from http://geneatlas.roslin.ed.ac.uk [19]. GRCh37.p13 is downloaded from https://www.ncbi.nlm.nih.gov/assembly/GCF_000001405.25/. The CADD scores are downloaded from https://cadd.gs.washington.edu [36]. The dbscSNC scores are downloaded from ftp://dbnsfp:dbnsfp@dbnsfp.softgenetics.com [37]. The GTEx eQTL data are downloaded from https://gtexportal.org/home/datasets [30]. The MAGMA tool can be found at https://ctg.cncr.nl/software/magma [29]. The Mouse Genome Informatics database is at http://www.informatics.jax.org. The MSigDB database is at https://www.gsea-msigdb.org/gsea/msigdb/index.jsp [32]. The MetamorphoSys software is downloaded from the UMLS after requesting a license and signing up for a UMLS Terminology Services (UTS) account, https://www.nlm.nih.gov/research/umls/index.html [26]. The ExAC database is at https://gnomad.broadinstitute.org [38]. All other data supporting the conclusions of this article are included in the main text and the additional files. All codes used for data preparations and analyses in this study are available at https://github.com/ZhaoXM-Lab/ukb-multimorbidity [61].
