# Supplementary material for: A global overview of genetically interpretable multimorbidities among common diseases in the UK Biobank
Source: Genome Med. 2021 Jul 5;13:110. doi: 10.1186/s13073-021-00927-6 (PMC8258962; doi:10.1186/s13073-021-00927-6)
Supplement: Supplementary file 1 — Additional file 1:. Supplementary Methods and Results. [file 13073_2021_927_MOESM1_ESM.docx]

**Supplementary Methods and Results**

**Multimorbidity relations among the whole set of 781 diseases**

For examining whether pre-selecting a subset of diseases with prevalence > 0.1% will affect the multimorbidity relations among common diseases or not, we further calculate the multimorbidity relations by focusing on the whole set of diseases (781 diseases). We find 12,895 multimorbidity relations among the whole set of diseases, in which 9,995 are multimorbidities among common diseases (Additional file 3: Table S12). The number of common-disease multimorbidities is reduced when using the whole set of diseases compared with that when using a subset of diseases (9,995<11,285), because the threshold of *P*-values decreases. Since the ICD10 codes that correspond to the same disease are merged into one disease by phecode [1], thus the common diseases (prevalence > 0.1%) are slightly different when considering the whole set and a subset of diseases. For example, J41, J42, J44 are merged into one disease when considering whole set of disease, but only J42, J44 are merged when considering a subset of diseases. This is because the J41 have prevalence < 0.1% and is removed when only considering diseases with prevalence > 0.1%. There are 373 diseases are commonly used. We find: (1) all the multimorbidities (7,762) obtained by using the whole set of diseases can be repeated by using a subset of diseases, and (2) 94.5% (7,762/8,218) multimorbidities obtained by using a subset of diseases are reproducible when using whole set of diseases. Therefore, the comparison result shows that no matter whether the whole set of diseases or only common diseases (prevalence > 0.1%) are considered, the multimorbidity connections among common diseases are almost the same.

**Directionality of the multimorbid disease-pairs**

We calculate the directionality between multimorbid disease-pairs by the method used by Jensen *et al* [2]. However, we find few multimorbidity relations are directional (1,034 multimorbidities are directional, and 10,251 are not). This may because the relatively small sample size of the UKB hospital inpatient data results in losing power to detect directionality of multimorbidity. Since few directional multimorbidities are observed, we choose to use both the directional and unidirectional multimorbidities for our main analysis, while keeping the directionality of multimorbidities available as a reference (Additional file 3: Table S2).

**Multimorbidity comparison with Blair *et al.* and Jensen *et al.* at different time windows**

We find nearly 30% (26099/96141) diseases-pairs with RR >1 and *P*-values < 6.4e-7 (Bonferroni correction threshold) (Additional file 2: Fig. S9). The diagnosis summary data in the UKB only includes about 0.4 million participants, which is relatively small compared with other electronic health record datasets (usually more than tens of millions) [3-5]. To reduce false positives, we identify multimorbidities by using time windows of 1 day, 0.5 year, 1 year, 2 years, 3 years, 4 years and 5 years, respectively, and then we compare the multimorbidities identified at different time windows with the Jensen’s and Blair’s results [2, 4].

Fig. S9 in Additional file 2 shows that the numbers of multimorbidities obviously decrease at the ‘1 day window’. Besides, from the Table S13 in Additional file 3, we can see that 1) the numbers of overlapping multimorbidities increase with more relaxed windows; 2) compared with Jensen *et al.*, the multimorbidity overlaps of all the windows are significant; 3) compared with Blair et al., the significance of the multimorbidity overlap gradually decreases as the time window increases, and the overlap obtained at the ‘1 day window’ is the most significant. These results suggest that using the ‘1 day window’ can help to reduce the false positives and get more reliable multimorbidities. Moreover, the ‘1 day window’ has an advantage of addressing the simultaneity in the definition of multimorbidity. We admit that some multimorbidities may be simultaneously diagnosed within few days or even dozens of days, and thus, in order to maintain this type of multimorbidities, we have selected multimorbid disease-pairs from those with more than 1% of patients diagnosed within 1 day instead of all patients diagnosed within 1 day. We argue that the more often two diseases are diagnosed within 1 day, the more likely they will be multimorbid. Therefore, in this study, we have used the multimorbidities identified at ‘1 day window’.

**Multimorbidity tendency of intra- and inter- categories based on new chapters**

We have further calculated the disease multimorbidity tendency of intra- and inter- categories based on the disease classification given by Zhou *et al* [6]. Zhou *et al.* classified 1,797 diseases into 17 distinct new chapters (NC01-NC17) by integrating phenotypic and molecular networks. The 1,797 diseases are based on ICD9 codes, thus we firstly map the ICD9 codes to ICD10 codes using the UMLS [7]. 1,209 ICD9 diseases are successfully mapped to 224 ICD10 diseases (Additional file 2: Fig. S10A), only covering 3,484 UKB multimorbidities —31% of all the identified multimorbidities in our analysis. By using the same method as described in main article (Multimorbidity tendency of intra- and inter-categories in Method), we only find the diseases within NC05 tend to co-occur (Additional file 2: Fig. S10B). NC05 includes 10 diseases (I05, I08, I10, I21;I22, I25, I35, I42, I46, G25, G95), mainly related to cardiovascular diseases. This multimorbidity tendency has been identified in our primary analysis based on physiological system-based disease classification. The failure to observe more multimorbidity patterns may be due to the incomplete coverage of diseases in the new chapters, as we see, only half of the common diseases is covered by the new chapters, and only about 31% of multimorbidities are included.

**References**

1. Wei WQ, Bastarache LA, Carroll RJ, Marlo JE, Osterman TJ, Gamazon ER, et al. Evaluating phecodes, clinical classification software, and ICD-9-CM codes for phenome-wide association studies in the electronic health record. PLoS One. 2017;12(7):e0175508. doi: <https://doi.org/10.1371/journal.pone.0175508>.
2. Jensen AB, Moseley PL, Oprea TI, Ellesoe SG, Eriksson R, Schmock H, et al. Temporal disease trajectories condensed from population-wide registry data covering 6.2 million patients. Nat Commun. 2014;5:4022. doi: <https://doi.org/10.1038/ncomms5022>.
3. Park J, Lee DS, Christakis NA, Barabasi AL. The impact of cellular networks on disease comorbidity. Mol Syst Biol. 2009;5:262. doi: <https://doi.org/10.1038/msb.2009.16>.
4. Blair DR, Lyttle CS, Mortensen JM, Bearden CF, Jensen AB, Khiabanian H, et al. A nondegenerate code of deleterious variants in Mendelian loci contributes to complex disease risk. Cell. 2013;155(1):70-80. doi: <https://doi.org/10.1016/j.cell.2013.08.030>.
5. Hidalgo CA, Blumm N, Barabasi AL, Christakis NA. A dynamic network approach for the study of human phenotypes. PLoS Comput Biol. 2009;5(4):e1000353. doi: <https://doi.org/10.1371/journal.pcbi.1000353>.
6. Zhou X, Lei L, Liu J, Halu A, Zhang Y, Li B, et al. A Systems Approach to Refine Disease Taxonomy by Integrating Phenotypic and Molecular Networks. EBioMedicine. 2018;31:79-91. doi: <https://doi.org/10.1016/j.ebiom.2018.04.002>.
7. Bodenreider O. The Unified Medical Language System (UMLS): integrating biomedical terminology. Nucleic Acids Res. 2004;32(Database issue):D267-70. doi: <https://doi.org/10.1093/nar/gkh061>.
