# Supplementary material for: A global overview of genetically interpretable multimorbidities among common diseases in the UK Biobank
Source: Genome Med. 2021 Jul 5;13:110. doi: 10.1186/s13073-021-00927-6 (PMC8258962; doi:10.1186/s13073-021-00927-6)
Supplement: Supplementary file 2 — Additional file 2: Supplementary Figures. Size distributions of the Reactome pathways, KEGG pathways, BioCarta pathways and PID pathways (top). Size distributions of disease and multimorbidity pathways (reactome and non-reactome) when only using pathways with size <= 200 (bottom) (Fig. S1). Correlation between the prevalence of diseases and the number of multimorbidities (Fig. S2). Splicing score of multimorbidity SNPs, other disease SNPs and non-disease SNPs (Fig. S3). Characteristics of the genetic components shared by multimorbidities when removing HLA-region variants (Fig. S4). pLIs of multimorbidity genes, other disease genes and non-disease genes when removing essential genes (Fig. S5). Degree distributions of diseases in multimorbidity networks that share loci and network level genetic components (Fig. S6). Multimorbidity overlaps interpreted by SNPs, genes, PPIs, pathways and genetic correlations (Fig. S7). Correlations among genetic correlation (rg), relative risk (RR), and phenotype similarity (PheSim) of multimorbidities (Fig. S8). Number of multimorbidities with time windows of 1 day, 0.5 year, 1 year, 2 years, 3 years, 4 years, 5 years and with no time limit (Fig. S9). Multimorbidity tendency of intra- and inter-categories based on the new chapters (NC) of diseases (Fig. S10). [file 13073_2021_927_MOESM2_ESM.docx]

|   **Fig. S1. Size distributions of the pathways. A** Size distributions of Reactome pathways, KEGG pathways, BioCarta pathways and PID pathways. **B** Size distributions of disease and multimorbidity pathways (reactome and non-reactome) by only using pathways with size <= 200 for enrichment analysis. |
| --- |
| 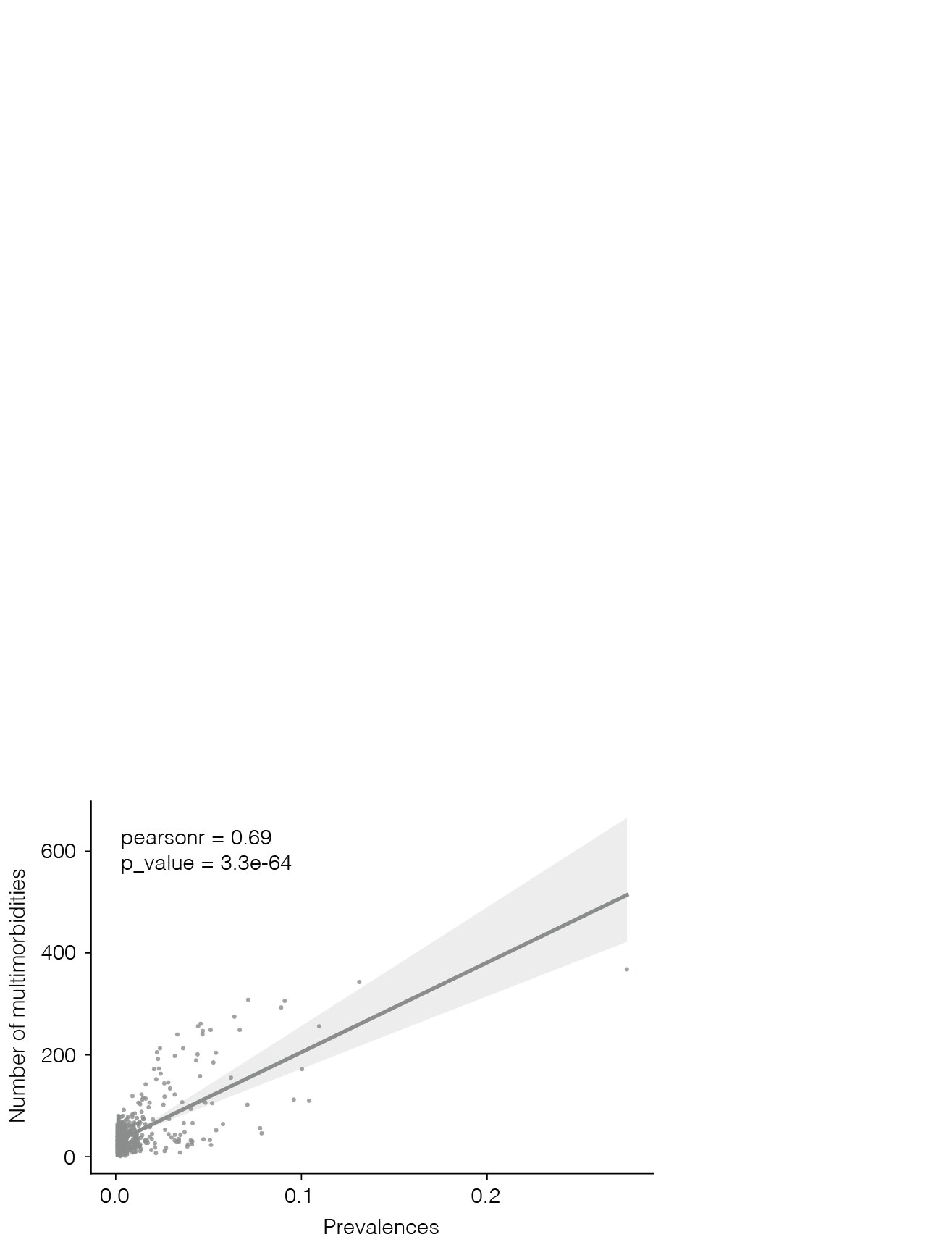  **Fig. S2. Correlation between the prevalence of diseases and the number of multimorbidities.** |

|   **Fig. S3. Splicing scores of SNPs.** **A** Splicing scores of non-disease SNPs, other disease-SNPs and multimorbidity-SNPs. **B** Splicing scores of non-disease SNPs, other disease-SNPs and multimorbidity-SNPs after removing HLA-region SNPs. |
| --- |
| 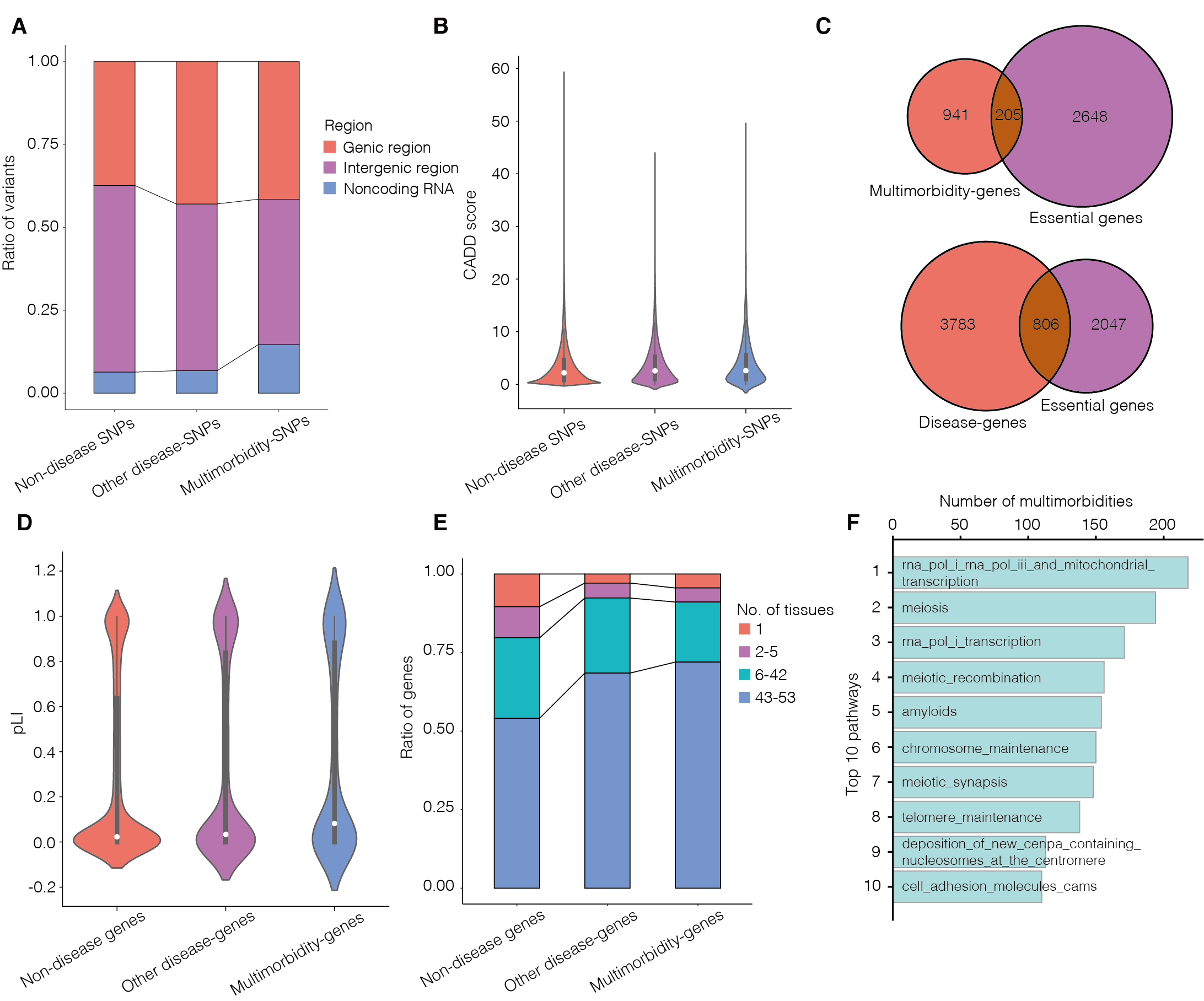  **Fig. S4. Characteristics of the genetic components shared by multimorbidities after removing HLA-region SNPs. A** The ratios of SNPs located in genic, intergenic, and noncoding RNA regions for multimorbidity-SNPs, other disease-SNPs, and non-disease SNPs. **B** CADD score distributions for multimorbidity-SNPs, other disease-SNPs, and non-disease SNPs. **C** Overlaps between multimorbidity-genes and essential genes, and between disease-genes and essential genes. **D** The pLI distributions of multimorbidity-genes, other disease-genes, and non-disease genes. **E** The ratios of genes expressed in certain numbers of tissues, for multimorbidity-genes, other disease-genes, and non-disease genes. **F** Top ten pathways that are shared by the largest numbers of multimorbidities. |
|   **Fig. S5. pLI scores of genes after removing essential genes. A** pLI scores of non-disease genes, other disease-genes and multimorbidity-genes. **B** pLI scores of non-disease genes, other disease-genes and multimorbidity-genes after removing HLA-region SNPs. |
|   **Fig. S6. Node (disease) degree distributions of the LG-network (A) and NG-network (B).** |
| 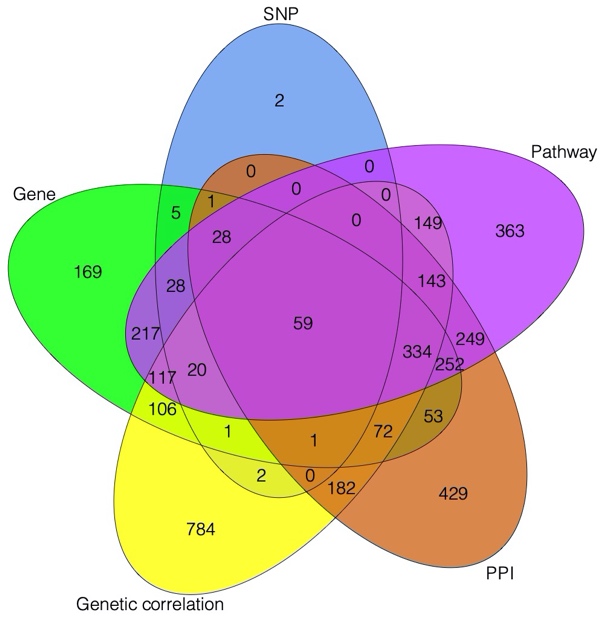  **Fig. S7.** **Multimorbidity overlaps interpreted by SNP, gene, PPI, pathway and genetic correlation.** |
| 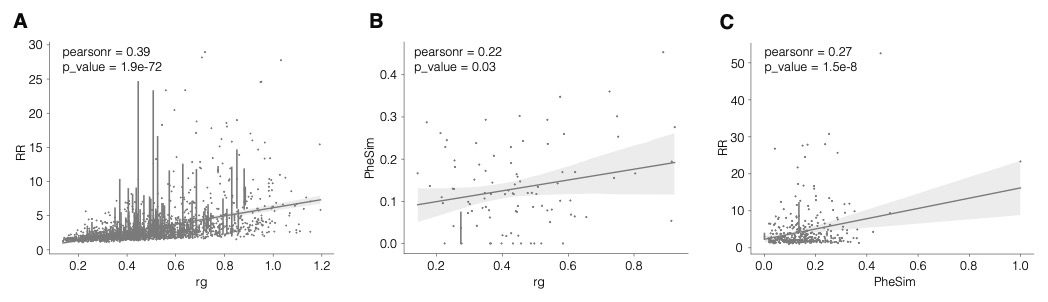  **Fig. S8. Correlations among genetic correlation (rg), relative risk (RR), and phenotype similarity (PheSim) of multimorbidities. A** Pearson correlation between genetic correlation (rg, x-axis) and relative risk (RR, y-axis). 6 dots with RR > 30 are removed for clarity of the figure. **B** Pearson correlation between genetic correlation (rg, x-axis) and phenotype similarity (PheSim, y-axis). Phenotype similarity was pre-calculated by van Driel *et al* [1]. 1 dot with PheSim > 0.6 is removed for clarity of the figure. **C** Pearson correlation between phenotype similarity (PheSim, x-axis) and relative risk (RR, y-axis). 1 dot with RR > 60 is removed for clarity of the figure.    **Fig. S9. Number of multimorbidities with time windows of 1 day, 0.5 year, 1 year, 2 years, 3 years, 4 years, 5 years and with no time limit. No time limit means that we do not use time window to preselect the disease-pairs for multimorbidity calculation.** |
|  |
|   **Fig. S10. Multimorbidity tendency of intra- and inter-categories based on new chapters (NC) of diseases given by Zhou *et al* [2]. A** The number of ICD10 diseases in each new chapter. **B** Disease multimorbidity tendency of intra- and inter-categories. Color and size of the circles represent the proportions of multimorbid relationships in all disease-pairs within a category or between two categories. The deeper the color and the larger the size of a circle, the higher the proportion is. Star represents adjusted P-value < 0.05 (FDR corrected). |

**References**

1. van Driel MA, Bruggeman J, Vriend G, Brunner HG, Leunissen JA. A text-mining analysis of the human phenome. Eur J Hum Genet. 2006;14(5):535-42. doi: <https://doi.org/10.1038/sj.ejhg.5201585>.
2. Zhou X, Lei L, Liu J, Halu A, Zhang Y, Li B, et al. A Systems Approach to Refine Disease Taxonomy by Integrating Phenotypic and Molecular Networks. EBioMedicine. 2018;31:79-91. doi: <https://doi.org/10.1016/j.ebiom.2018.04.002>.
